# Therapeutic induction of high endothelial venules (HEVs) to enhance T-cell infiltration in tumors

**DOI:** 10.18632/oncotarget.22276

**Published:** 2017-11-03

**Authors:** Elizabeth Allen, Rindert Missiaen, Gabriele Bergers

**Affiliations:** Gabriele Bergers: Department of Oncology, Laboratory of Tumor Microenvironment and Therapeutic Resistance, VIB-Center for Cancer Biology, Katholieke Universiteit Leuven, Leuven, Belgium

**Keywords:** cancer, antiangiogenic therapy, immunotherapy, high-endothelial venules, lymphotoxin-beta receptor

There has been an explosion of clinical trials in an increasing variety of tumors utilizing immunotherapies as the consequence of their striking efficacy in the treatment of certain cancers, albeit in a minor population of patients [1]. In parallel, years of clinical trials with antiangiogenics demonstrated encouraging results in a small subset of patients, but have failed to produce increased overall survival [2]. Accumulating evidence indicates that a crosstalk exists between angiogenesis and immunity - an aberrant angiogenic tumor vasculature can thwart the immune response by reducing immune T effector cell infiltration into tumors, along with leukocyte-endothelial transmigration and extravasation [[3, 4] and references therein]. In line with these observations, our group and the DePalma group [3, 4] found that targeting the tumor vasculature can transiently prune and normalize tumor vessels during a response phase, leading to an angiostatic phenotype characterized by increased infiltration of immune-stimulatory cells and tumor stasis (Figure 1A)[5]. During relapse, however, tumors initiated an adaptive immune suppressive mechanism that limited the efficacy of antiangiogenic agents by upregulating the negative immune checkpoint regulator, programmed cell death ligand 1 (PD-L1) in tumor and stromal cells. This lead to immunosuppression triggered by PD-L1 binding PD-1 on the surface of activated T cells to produce T cell anergy or exhaustion [3, 4].

**Figure 1:**
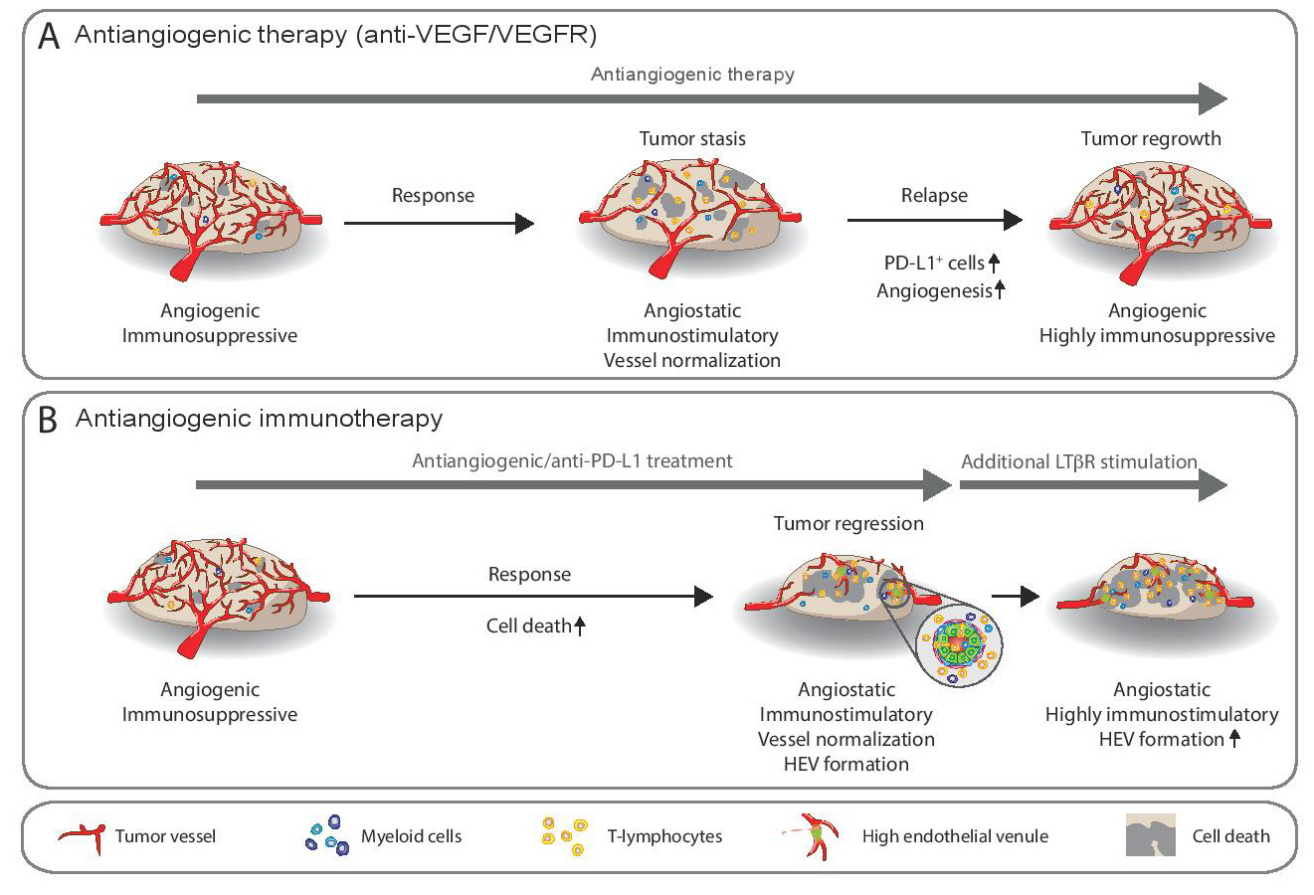
**A.** Tumors create an immunosuppressive tumor microenvironment characterized by the presence of myeloid cells and a tumor vasculature with angiogenic and immunosuppressive properties. Antiangiogenic therapy transiently reverts this phenotype by vessel normalization and repolarization of angiostatic and immune stimulatory myeloid cells and enhanced influx of CTLs to produce tumor stasis. Following the response phase, tumors initiate mechanisms to reinstate an immunosuppressive environment involving the innate and adaptive immune system. Thereby, CTLs secrete INFγ that upregulates PD-L1 in various cell types while tumor –secreted factors activate myeloid cells to render them angiogenic, immunosuppressive and non-responsive to antiangiogenic therapy. **B.** Combination of antiangiogenic/anti-PD-L1 therapy can substantially extend the response phase to produce tumor regression consequent to increased cell death, and an angiostatic/immune stimulatory microenvironment when HEVs are induced that enhance CTL- influx; additional LTβR stimulation using an agonistic antibody can further increase HEV formation and infiltration of activated CTLs.

Combining immunotherapy using anti-PD-L1 with antiangiogenic therapy had reciprocally beneficial effects in that immunotherapy targeted evasion from antiangiogenic therapy, while vascular normalization elicited by antiangiogenic treatment could increase lymphocyte infiltration and activation [3, 4]. In addition, antiangiogenic immunotherapy produced an unanticipated response – it induced a specialized form of blood vessels in treated tumors, reminiscent of high endothelial venules (HEVs) [4], that are typically found in lymphoid tissue.

HEVs are morphologically and functionally distinct postcapillary venules that mediate the adhesion and transendothelial migration of circulating T- and B-cells to secondary lymphoid organs, including lymph nodes, where they encounter antigens and become activated [[6] and references therein]. HEVs can also be induced at sites of chronic inflammation found in autoimmune diseases or infection and become an integral part of tertiary lymphoid structures (TLSs) when they are surrounded by compartimentalized immunestimulatory immune cells (naive and CD8+ GranzymeB+ T-cells and others). While under these circumstances, the development of TLSs is thought to exacerbate disease, the spontaneous development of HEVs observed in several cancers has been found to be associated with improved patient outcomes. [[7] and references therein]. Taken together, these results suggest that therapeutic HEV induction in tumors may enable potent T-cell infiltration in human tumors to overcome and reinvigorate a rate-limiting step in the cancer-immunity cycle.

Several lines of evidence point to a critical role of lymphotoxin (LIGHT, LTαβ) /lymphotoxin β-receptor (LTβR) signaling in HEV formation, and recent studies revealing that EC-specific LTβR knockout animals lack fully functional HEVs, reinforce this notion [[7] and references therein]. Thereby, lymphotoxin-expressing CD11c+ dendritic cells (DCs) play a crucial role in maintenance of differentiated HEVs, since CD11c depletion disrupts HEV structure and function which can be restored by DC infusion [8]. The LTβR pathway appears to be important for HEV formation in tumors as well because lymphotoxins were upregulated in tumors undergoing antiangiogenic immunotherapy concomitant with a LTβR -NFκB gene signature. Importantly, full activation of LTβR in tumors using an agonistic antibody was sufficient to substantially enhance HEV formation during antiangiogenic immunotherapy and induce HEV in recalcitrant GBM to sensitize tumors to antiangiogenic PD-L1 therapy, while combination treatment with LTβR antagonists reversed these effects [4].

While tantalizing, these results raise some additional questions regarding optimization of therapeutic regimens, including those related to the stability of therapeutically induced HEVs, and whether the composition of immune infiltrates will change over time. While systemic activation of LTβR may not be suitable for the clinic due to potential immune-related side effects, the concept of therapeutic HEV induction nevertheless holds great promise to markedly improve immune responses to tumors when used in parallel with many approved immunotherapies and vascular-normalizing antiangiogenics, since naive T-cells can be locally recruited and activated by cytokines or dendritic cells within the tumor without being diluted in transit from the lymph node through the bloodstream.

## References

[1] Champiat S (2017). Clin Cancer Res.

[2] Ferrara N (2016). Nat Rev Drug Discov.

[3] Schmittnaegel N (2017). Sci Transl Med.

[4] Allen E (2017). Sci Transl Med.

[5] Rivera L (2015). Cell Reports.

[6] Ager A (2015). OncoImmunology.

[7] Martinet L (2013). OncoImmunology.

[8] Moussion C (2011). Nature.

